# Draft Genome Sequences of 11 *Salmonella enterica* Strains with Variable Levels of Barotolerance

**DOI:** 10.1128/genomeA.00952-16

**Published:** 2016-09-22

**Authors:** Jennifer Ronholm, Nicholas Petronella, Sandeep Tamber

**Affiliations:** a Microbiology Research Division, Bureau of Microbial Hazards, Food Directorate, Health Products and Food Branch, Health Canada, Ottawa, Ontario, Canada; b Biostatistics and Modelling Division, Bureau of Food Surveillance and Science Integration, Food Directorate, Health Products and Food Branch, Health Canada, Ottawa, Ontario, Canada

## Abstract

The diversity of the genus *Salmonella* is reflected in the physiological adaptations used by its members in response to stressors such as high pressure. Here we report the draft whole genome sequences of 11 *Salmonella enterica* strains, five sensitive strains and six demonstrating high levels of pressure resistance.

## GENOME ANNOUNCEMENT

The gastrointestinal pathogen *Salmonella enterica* can survive on a variety of food commodities, including ready-to-eat foods. This category of food is generally not subject to any cooking steps that can inactivate bacterial pathogens. High-pressure processing is a nonthermal form of food preservation using pressure ranging from 100 to 800 MPa to inactivate bacteria and other spoilage organisms (1). We have initiated a study investigating the responses of diverse *Salmonella* isolates to high pressure. During the course of the investigation, we identified a subset of strains that exhibited unusually high levels of resistance to pressures of 600 MPa. Here we report the draft whole-genome sequences of six barotolerant *S. enterica* strains and five sensitive strains.

The strains are housed in Health Canada’s Bureau of Microbial Hazards *Salmonella* strain collection. Genomic DNA was isolated from 2 to 3 colonies of a freshly prepared pure culture on tryptic soy agar with a Maxwell 16SEV cell DNA purification kit (Promega, Madison, WI). Sequencing libraries were prepared using a Nextera XT DNA sample preparation kit (Illumina, San Diego, CA) and read using a benchtop Illumina MiSeq instrument for 300 cycles. Reads were assembled into nonoverlapping contiguous sequences *de novo* using SPAdes version 3.5.0 (2), the Mismatch Corrector tool and BayesHammer for error correction (3). These procedures resulted in high-quality draft genome sequences for each of the 11 strains (Table 1). Gene predictions and annotations were performed by the National Center for Biotechnology Information (NCBI) Prokaryotic Genome Annotation Pipeline (PGAP) (4). Sequence types and eBURST groups were assigned according to the multilocus sequence typing (MLST) criteria described in Achtman et al. (5).

**TABLE 1:** Sequencing and MLST results of 11 *S. enterica* strains with variable levels of barotolerance

Strain	Biosample no.	Accession no.	Genome coverage (fold)	Genome size (bp)	No. of contigs	G+C content (%)	Sequence type (eBURST group)
SI01	SAMN05356920	MATB00000000	22.97	4,751,976	66	52.20	ST32 (eBG31)
SI02	SAMN05356921	MATC00000000	25.67	4,587,815	38	52.30	ST32 (eBG31)
SMo01	SAMN05356922	MATD00000000	32.81	4,670,235	30	52.17	ST316 (eBG40)
SMo02	SAMN05356923	MATE00000000	44.34	4,607,463	25	52.25	ST4 (eBG40)
SMu01	SAMN05356924	MATF00000000	53.83	4,631,312	19	52.20	ST112 (eBG8)
SMu02	SAMN05356925	MATG00000000	42.89	4,733,682	36	52.03	ST82 (eBG8)
STh01	SAMN05356926	MATH00000000	26.84	4,758,749	34	52.24	ST26 (eBG28)
STh02	SAMN05356927	MATI00000000	58.45	4,752,155	30	52.14	ST26 (eBG28)
STy01	SAMN05356928	MATJ00000000	47.81	4,965,918	63	52.12	ST19 (eBG1)
STy02	SAMN05356929	MATK00000000	125.05	4,808,032	53	52.14	ST19 (eBG1)
STy03	SAMN05356930	MATL00000000	127.16	4,808,325	49	52.13	ST19 (eBG1)

### Accession number(s).

These sequences have been deposited in DDBJ/ENA/Genbank under the accession numbers provided in Table 1. The versions described in this paper are the first versions, MATB01000000 to MATL01000000.
